# Hybridization between *Felis silvestris silvestris* and *Felis silvestris catus* in two contrasted environments in France

**DOI:** 10.1002/ece3.5892

**Published:** 2019-12-02

**Authors:** Marie‐Pauline Beugin, Olivier Salvador, Guillaume Leblanc, Guillaume Queney, Eugenia Natoli, Dominique Pontier

**Affiliations:** ^1^ Laboratoire de Biométrie et Biologie Evolutive UMR5558 CNRS Univ Lyon Université Lyon 1 Villeurbanne France; ^2^ Animal Genomics Laboratory ANTAGENE La tour de Salvagny France; ^3^ Réserve naturelle nationale de Jujols et de Nohèdes Réserves Naturelles Catalanes Prades France; ^4^ Association LOANA Lorraine Association Nature Champougny France; ^5^ Canile Sovrazonale ASL Rome 3 Rome Italy

**Keywords:** *F. s. catus*, *Felis silvestris silvestris*, hybridization, microsatellites, noninvasive sampling, relatedness

## Abstract

European wildcat (*Felis silvestris silvestris*) populations are fragmented throughout most of the whole range of the subspecies and may be threatened by hybridization with the domestic cat *F.s. catus*. The underlying ecological processes promoting hybridization remain largely unknown. In France, wildcats are mainly present in the northeast and signs of their presence in the Pyrenees have been recently provided. However, no studies have been carried out in the French Pyrenees to assess their exposure to hybridization. We compared two local populations of wildcats, one living in a continuous forest habitat in the French Pyrenees, the other living in a highly fragmented forest‐agricultural landscape in northeastern France to get insights into the variability of hybridization rates. Strong evidence of hybridization was detected in northeastern France and not in the Pyrenees. Close kin in the Pyrenees were not found in the same geographic location contrary to what was previously reported for females in the northeastern wildcat population. The two wildcat populations were significantly differentiated (*F*_ST_ = 0.072) to an extent close to what has been reported (*F*_ST_ = 0.103) between the Iberian population, from which the Pyrenean population may originate, and the German population, which is connected to the northeastern population. The genetic diversity of the Pyrenean wildcats was lower than that of northeastern wildcat populations in France and in other parts of Europe. The lower hybridization in the Pyrenees may result from the continuity of natural forest habitats. Further investigations should focus on linking landscape features to hybridization rates working on local populations.

## Graphical abstract

Hybridization in the French Pyrenees has never been assessed. Our study provides the first genetic characterization of a local population of European wildcats in the French Pyrennees and shows that hybridization remains limited in this area. The Pyrenean population was significantly differentiated from the North‐eastern population of wildcats also included in the study.

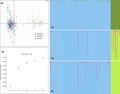

## 1 INTRODUCTION

Hybridization is a common phenomenon in nature (Abbott et al., 2013; Mallet, 2005), particularly between subspecies, that is due to incomplete reproductive isolation and therefore a higher likelihood of successful interbreeding (Levin, 2002; Randi, 2008; Rhymer & Simberloff, 1996). The role of hybridization in conservation represents a dilemma. Whereas natural hybridization is recognized as a major evolutionary process involved in adaptive dynamics and the maintenance of biodiversity (Arnold, 1992; Qi, Lu, Gao, Hu, & Fu, 2014), anthropogenic hybridization—that is hybridization facilitated by human activities (alteration of habitats and populations)—is widely perceived as a potential threat for a wide range of animal species (Allendorf, Leary, Spruel, & Wenburg, 2001; Bohling, 2016; Genovart, 2008). The resulting elimination of barriers between otherwise distinct populations may lead to introgression and up to genomic extinction due to loss of evolutionary adaptation (Allendorf et al., 2001; Rhymer & Simberloff, 1996). Some hybridization cases raise even more problems than others, notably when hybridization occurs between domesticated animals and their closely wild relatives. The introgression of alleles from domesticated populations can indeed decrease fitness in the wild by disrupting important adaptations, threatening the genetic integrity of the wild species (Randi, 2008).

Anthropogenic hybridization is a major concern for the European wildcat *Felis silvestris silvestris*, a medium‐sized carnivore that is widely spread across Europe from the Iberian Peninsula to the Caucasus Mountains and northwards up to Scotland (Hertwig et al., 2009; Mattucci, Oliveira, Lyons, Alves, & Randi, 2016; O'Brien et al., 2008; Oliveira, Godinho, Randi, & Alves, 2008; Pierpaoli et al., 2003; Witzenberger & Hochkirch, 2014), with a continental distribution that is largely fragmented both at regional and local scales (Mattucci et al., 2016). The species is threatened over its entire distribution area by its closely related domestic counterpart *F. s. catus* (Yamaguchi, Kitchener, Driscoll, & Nussberger, 2015). Free‐ranging domestic cats are often present in much higher density than wildcats, which creates the conditions for crossbreeding and introgression of domestic alleles into the wildcat genome. However, studies across the area of distribution of the European wildcat have shown that there is a high degree of variability in the extent of admixture with domestic cats. High levels (up to 45% corresponding to the proportion of hybrids—F1, backcrosses, depending on the study—in a population of wildcats) of hybridization have been reported in Hungary and Scotland (Beaumont et al., 2001; Daniels et al., 2001; Lecis et al., 2006; Pierpaoli et al., 2003; Randi, 2008), while lower levels (between 0% and 2%) of interbreeding with domestic cats have been shown in Germany, Italy, Spain, and Portugal (Lecis et al., 2006; Oliveira et al., 2008; Pierpaoli et al., 2003; Randi, Pierpaoli, Beaumont, Ragni, & Sforzi, 2001). Direction of the gene flow also varied, some studies reporting a gene flow from domestic cats to wildcats (Nussberger, Wandeler, Weber, & Keller, 2014; Oliveira, Godinho, Randi, Ferrand, & Alves, 2007) while others showed the opposite with a detected flow from wildcats to domestic cats (Hertwig et al., 2009). The causes of such a high degree of heterogeneity in hybridization modalities and subsequent introgression are not known, but different environmental conditions (e.g., habitat fragmentation and urban pressure), ecological barriers (Gil‐Sànchez, Jaramillo, & Barea‐Azcòn, 2015), relative numbers of wild and domestic cats, or population histories (Crispo, Moore, Lee‐Yaw, Gray, & Haller, 2011; Mattucci et al., 2019; Pierpaoli et al., 2003) could all play a role. Local investigations of threats are thus required to identify conservation problems and design the best options for wildcat conservation (Lozano & Malo, 2012; Mattucci et al., 2019).

In France, hybridization between *Felis* subspecies has been investigated at different spatial scales in the northeast (Germain, Benhamou, & Poulle, 2008; Germain, Ruette, & Poulle, 2009; O'Brien et al., 2009; Say, Devillard, Léger, Pontier, & Ruette, 2012) with substantial rates of hybridization close to 25% on average. In Beugin, Leblanc, Queney, Natoli, and Pontier (2016), a much lower rate was found (2.3%). This difference in the rate of hybridization may result from different sampling strategies: previous studies were led exclusively on road‐killed animals while Beugin et al. (2016) relied mostly on live‐trapped animals (32 out of 42). If hybrids tend to live in intermediary environments as suggested by Germain et al. (2008, 2009), they may be more represented within roadkills as roads are located in proximity to villages, and thus between urban and wild environments. Sampling individuals within the habitat of domestic cats (villages) and wildcats may thus be crucial to truly understand hybridization patterns. In France, wildcats are also observed in the Pyrenees. However, the French Pyrenean populations, which are suspected to be relatively isolated within the species' distribution range in France and northern Europe (Mattucci et al., 2016; Say et al., 2012), have not been thoroughly studied and much remained to be investigated regarding this population. In particular, its connection with the northeastern population or with Spanish population of European wildcats (Mattucci et al., 2016; Say et al., 2012), and the extent to which they hybridize with domestic cats are not known.

In this study, we aim to compare hybridization patterns of two local populations living in two contrasted environments, working mostly on samples collected within the habitat of wildcats, either blood or hair from live‐trapped individuals or feces. We extend our knowledge on the northeastern populations of wildcats and domestic cats studied in Beugin et al. (2016) by integrating a much larger sample of domestic cats; this allows us to better predict the rate of hybridization within the domestic cats and to assess whether hybrids are more present in villages than in forests, a pattern that we expect to be promoted by the sexual segregation—males at the periphery of the forest, females within—reported in Beugin et al. (2016) and Oliveira et al. (2018). In addition, we sampled and analyzed genetic data in a French Pyrenean wildcat population. In this area, the forest landscape is highly continuous, contrary to the fragmented forests of northeastern France where patches of forest are intermixed with agriculture fields (Cemagref, Chéry, & Deshayes, 2010) like in Hungary. Given the continuous forest habitat with few interfaces between forests and villages in this area, we may expect hybridization to be rare or absent in Pyrenean European wildcats.

## 2 MATERIAL AND METHODS

### 2.1 Study areas and sampling strategies

**Figure 1:**
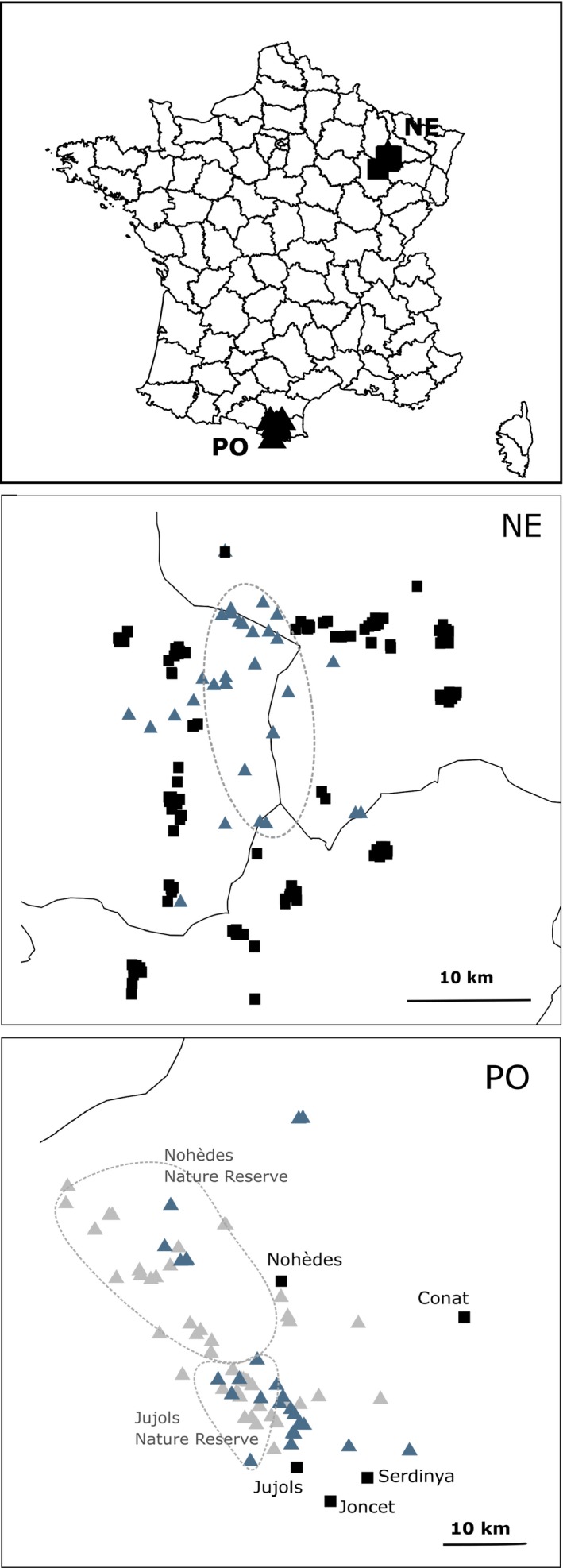
Localization of the study areas in the Pyrenees and northeastern France. The triangles represent all the locations where feces, camera‐trapping or direct observations attested for the presence of the European wildcat. Blue triangles correspond to locations where European wildcats' samples were taken and genotyped. The black squares correspond to locations where domestic cats were sampled. Ellipses correspond to the forested area in northeastern France (NE) and to the nature reserve in Pyrénées Orientales (PO)

**Figure 2:**
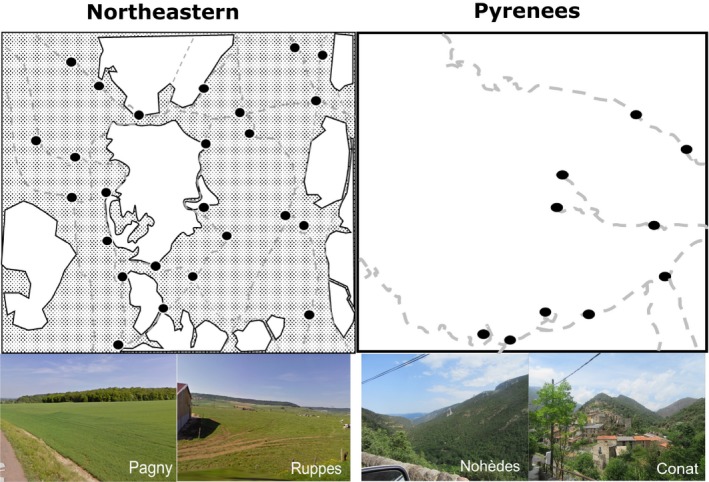
Differences between the two areas of study. White areas represent forests, gray areas represent urban areas and agricultural fields. Roads are represented by gray dotted lines. Villages are represented by black filled circles. The pictures have been taken on the field to illustrate landscape differences

In northeastern France (NE), the area of study covered approximately 400 km^2^ (Beugin et al., 2016). The landscape is substantially fragmented (Cemagref et al., 2010) and consists of an alternation between forests, agricultural fields, and permanent grass with elevations ranging from 250 to 400 m. A total of sixteen villages (30 to 600 inhabitants per village) were in direct proximity with the forest where wildcats were sampled (Figures 1 and 2). In the Pyrenees (PO), the Nohèdes Nature Reserve presents elevations ranging from 760 to 2,459 m while the elevation of the Jujols Nature Reserve ranges between 1,100 and 2,172 m. The study area covers a total surface of 325 km^2^ of continuous forest (oak, maple, ash, pines, beech). These two nature reserves are in proximity with 10 villages (30–230 inhabitants per village), and more particularly with the village of Nohèdes, Urbanya, Conat and Jujols (see Figures 1 and 2).

In northeastern France, domestic cats (*N* = 371) and wildcats (*N* = 32) were captured (Figure 1). Wildcats were caught in a wooden cage trap (60 × 60 × 120 cm), with a single sliding door, containing crushed valerian roots (*Valeriana officinalis*), a common attractant for cats (Jerosch, Götz, Klar, & Roth, 2010; Monterroso, Alves, & Ferreras, 2011). Individuals were assigned in the field to each subspecies according to morphological features (body weight, tarsus length, tail shape, pelage color, dorsal line, flank stripes); individuals presenting features characteristic of the European wildcat were assigned as such. Other individuals were assigned as domestic cats. Blood samples were collected for each individual and stored at −20°C, from November to February of each year between 2008 and 2011. Trapped individuals were anaesthetized with ketamine chlorohydrate (Imalgène 1000, 15 mg/kg, Merial) and acepromazine (Vetranquil 5.5%, 0.5 mg/kg, Ceva). A permanent subcutaneous electronic device (transponder Trovan, AEG & Telefunken Electronic) was injected in each cat under the skin on the shoulder to aid subsequent identification. In addition, ten wildcat samples were obtained on road‐killed individuals, which brings the total count of northeastern wildcats to 42. The fieldwork has been conducted by qualified people (trained ecologists) according to current French legislation. Accreditation has been granted to the UMR‐CNRS5558 (accreditation number 692660703). It encompasses the capture and anesthesia of cats, and blood collection. Individuals were monitored until they had fully recovered from the anesthesia. None presented any adverse effect following this work. In the eastern part of the French Pyrenees, fresh feces of wildcats were collected opportunistically from 2010 to 2016 in the nature reserves of Jujols and Nohèdes (see Figure 1). Experienced field agents from the nature reserves have collected evidence for additional occurrences of European wildcats since 1993 based on camera‐trapping surveys, direct observations or feces, which has allowed us to associate the sampling of fresh feces to the overall presence of the European wildcat in the study area (e.g., Lozano, Virgós, & Cabezas‐Díaz, 2013). For domestic cats, hairs were sampled in 2010 and 2017 in the villages of Nohèdes (*N* = 20), Conat (*N* = 4), and Serdinya (*N* = 3), located on the edge of the reserves (see Figure 1). Only individuals born in the villages, sterilized or not, were included in our sampling. A total of 71 feces and 27 hair samples were collected. Feces were sampled in individual plastic bags and kept at −20°C; and hairs in dry closed envelopes. The two populations were sampled over different time frames. No major changes were observed in the two areas of study between 2010 and 2017. We have thus no reason to think that the difference in time frames impacted our results.

### 2.2 DNA extraction and microsatellite genotyping

All individuals were genotyped using 22 autosomal microsatellite markers (Table S1). The sex of individuals was determined using the zinc‐finger regions of the X and Y chromosomes (Pilgrim, Mckelvey, Riddle, & Schwartz, 2005, Table S1). Blood samples (northeastern France samples) were genotyped only once. For noninvasive samples, a first genotyping was conducting on all samples. Those for which none or less than five loci were amplified were discarded, the other samples were genotyped one to three additional times (hereafter referred to as repeats). A consensus genotype was built to obtain a genotype per sample. A quality index (referred to as QI thereafter, Miquel et al., 2006) was calculated in order to assess the quality of the samples. For each locus and each repeat, the genotype obtained was compared to the consensus genotype built over all repeats. A repeat where the consensus genotype is obtained is rated with QI equal to 1, a repeat corresponding to a homozygote genotype while the consensus genotype is heterozygote will have a QI equal to 0.5, and a missing data will correspond to QI equal to 0. The QI are then averaged over all repeats for each locus, and then over all loci for an individual to obtain a QI per sample. Only individuals presenting a QI superior than 0.6 were included in the subsequent analyses.

Consensus genotypes were built as follows. Two genotypes were considered to represent the same individual when (a) they were identical, (b) they only differed by missing data and these missing data did not represent more than ten microsatellite markers, and (c) they differed by a single difference that could be explained by allelic dropout.

### 2.3 Subspecies characterization

We used the computer program STRUCTURE v 2.3.4 (Falush, Stephens, & Pritchard, 2003; Pritchard, Stephens, & Donnelly, 2000) and Evanno's method (Evanno, Regnaut, & Goudet, 2005) to determine the number of clusters *K* describing the highest level of differentiation in our populations using STRUCTURE Harvester. We ran STRUCTURE for *K* varying between 1 and 5 (10 iterations for each value of *K*) using the *admixture model* with correlated alleles frequencies for 100,000 burn‐in iterations and 300,000 MCMC iterations. For each of the *K* clusters, we conducted a similar STRUCTURE analysis (with values of *K* ranging from 1 to 5) to further investigate how genetic diversity was partitioned within each cluster. STRUCTURE clustering analysis relies on strong assumptions including Hardy–Weinberg equilibrium (Pritchard et al., 2000). As this assumption may not be respected in the case of the wild cat and domestic cat populations, clustering was also performed using a principal component analysis (PCA) implemented in the R packages ade4 (Dray & Dufour, 2007). Contrary to STRUCTURE algorithm, this approach does not rely on any specific population genetic model.

Deviations of loci from both Hardy–Weinberg equilibrium (HWE) and linkage equilibrium were both tested using FSTAT v 2.9.3.2. (Goudet, 1995) with a Bonferroni correction for each population of wildcats and domestic cats. Loci showing a departure from HWE were discarded from the analysis. For each locus, the frequency of null alleles was assessed following Brookfield's method (Brookfield, 1996), and its impact on a possible deviance from HWE tested using binomial tests according to de Meeüs, Béati, Delaye, Aeschlimann, & Renaud (2002). All loci exhibiting significant evidence of null alleles causing deviation from HWE were discarded from further analyses. The software Genetix 4.05.2 (Belkhir, Borsa, Chikhi, Raufaste, & Bonhomme, 1996) was used to assess Weir and Cockerham's pairwise *F*_ST_ between wildcat and domestic cat populations. A thousand permutations were run to assess the significance of pairwise *F*_ST_. We used the function AMOVA in the R package pegas (Paradis, 2010) to test whether the subspecies and the geographical area significantly explained the variability observed between populations.

In order to compare allelic richness and heterozygosities between populations and subspecies, we randomly selected 18 individuals in each population (which corresponded to the entire sample for Pyrenean wildcats) and determined the number of alleles per locus for each population. We repeated the sampling procedure 100 times and calculated overall repeats the mean number of alleles per locus, and the expected and observed heterozygosities using adegenet (Jombart, 2008).

### 2.4 Spatial structure and relatedness

The 52 fresh fecal samples for which the sampling location was recorded were displayed on a map using QGIS v2.8.1. (Quantum GIS Development Team, 2012), together with the other indices of presence of European wildcats (feces and photo trapping). The program ML‐Relate (Kalinowski, Wagner, & Taper, 2006) was used to calculate pairwise relatedness between all wildcat individuals. The coefficient *r* corresponds to the probability for each locus that individuals share zero, one or two alleles that are identical by descent. Using a linear model, we tested whether sex or relatedness were a significant predictor of the pairwise geographical distance between individuals. Geographical distances between individuals were calculated with QGIS v2.8.1. We considered the mean pairwise distance between samplings for individuals sampled several times. Statistical analysis was performed in R 3.3.3. The same procedure was conducted in northeastern France considering the trapping location or location where the body was found, to establish geographical distance.

### 2.5 Admixture analyses

We combined several computer programs and approaches to determine which individuals were most probably hybrids.

Firstly, we used the outputs (mean probabilities of assignment, referred to as *q*‐values thereafter, and 90% credibility intervals) of the STRUCTURE analysis described before, which we called “general analysis.” Considering the genetic differentiation between wildcats and domestic cats (*F*_ST_ between 0.1 and 0.2, Lecis et al., 2006; Oliveira et al., 2008; Mattucci et al., 2013), we expected first‐generation backcrosses and parental individuals to have overlapping *q‐*values distribution. In order to take into account this limitation and provide a range for the rate of hybridization, we used two approaches to detect hybrids: a “conservative” approach that tends to underestimate the number of hybrids, and a “relaxed” approach that tends, on the contrary, to over‐estimate their proportion (Figure S2). With the conservative approach, all individuals from the resampled dataset with a *q*‐value lower than the thresholds were detected as hybrids; with the relaxed method, all individuals from the resampled dataset with a 90% credibility interval lower than the threshold were detected as hybrids, all individuals above the threshold were considered as parental individuals. For this first detection step, we considered a threshold of 0.8, which has been widely used in the literature to detect hybrids and notably hybrids between *Felis* subspecies (Le Roux, Foxcroft, Herbst, & MacFadyen, 2015; Lecis et al., 2006; Mattucci et al., 2013). Rohde, Hau, Weyer, and Hochkirch (2015) showed that a 80% threshold does not only avoid overestimating the number of hybrids, but also minimizes the general number of mis‐assignments.

Secondly, we used a “resampling” approach to identify hybrids, which had two advantages: (a) It removes possible biases due to the much higher number of domestic cats sampled compared to European wildcats (Puechmaille, 2016); (b) it allows to assess a confidence interval for the rate of hybridization. We randomly selected 70 domestic cats (which allows each domestic cat to be sampled more than three times with a limited number of resamplings) out of the 371 domestic cats sampled in northeastern France. We built a dataset (hereafter referred to as “resampled dataset”) with these 70 individuals, the 42 wildcats from northeastern France, and all Pyrenean wildcats and domestic cats. We ran STRUCTURE on this resampled dataset (burn‐in length of 10,000; MCMC chain length of 30,000; admixture model), and we used all the individuals with a *q*‐value higher than 0.9 to simulate parental individuals (200 individuals for each subspecies) and hybrids (30 F1, 30 F1*wildcats and 30 F1*domestic cats) using the function *Hybridize* from adegenet. We ran STRUCTURE with the same parameters as before on these simulated individuals, and we used the lowest *q*‐value reached by simulated parental individuals as threshold to detect hybrids in the resampled dataset. Again, we detected hybrids both with the conservative and relaxed approach. We built 30 resampled datasets, which allowed us to sample each northeastern domestic cat 5.66 times on average (*SD* = 2.10). Confidence intervals (95%) were determined on the basis of these 30 repeats. On the individual level, we considered that an individual is presenting signs of hybridization if it is detected as hybrid in at least 15 samplings.

Finally, we used two other methods: NewHybrids (Anderson & Thompson, 2002) and Snapclust (Beugin, Gayet, Pontier, Devillard, & Jombart, 2018). For NewHybrids, we performed 100,000 burn‐in and 100,000 MCMC iterations including F1, F2, and first‐generation backcrosses in the analysis. Snapclust tends to detect more easily individuals as hybrids than NewHybrids (Beugin et al., 2018). Given that we do not expect first‐generation backcrosses to be perfectly distinguishable from parents with the panel of 22 microsatellite markers, we chose to include only F1 to avoid noninterpretable noise.

Additionally, the direction of the gene flow between wild and domestic cats was assessed by estimating the rate of contemporary migration per generation (within the last three generations) with the computer program BayesAss 3.0.3. (Wilson & Rannala, 2003) with a MCMC chain of 5,000,000 after a burn‐in period of 1,000,000 with a sampling interval of 2,000. Different mixing parameters were tested (migration rate: 0.01–0.2–0.5, inbreeding coefficient: 0.1–0.2–0.5, allele frequency: 0.1–0.2–0.5) to check the reliability of the results obtained. We used the program Tracer 1.7.1 (Rambaut, Drummond, Xie, Baele, & Suchard, 2018) to assess the effective sample size (ESS).

## 3 RESULTS

### 3.1 Amplification success and characterization of the populations

Feces and/or direct observations of European wildcats have been reported all over the nature reserves of Jujols and Nohèdes up to an elevation of 2,430 m. Genotyped samples were collected over the entire area where signs of the presence of the European wildcat had been reported (see Figure 1). Forty‐four out of the 71 fresh feces collected, and 22 out of the 27 hair samples collected showed a QI > 0.6 (mean = 0.84, *SD* = 0.12, see Figure S3). On average, 74.1% (*SD* = 0.31) of the loci were successfully amplified using feces while 81.5% (*SD* = 0.32) of the loci were successfully amplified on average from hairs.

We identified 39 unique genotypes including 21 domestic cats (15 females and 6 males) and 18 European wildcats (10 females and 8 males). Six (3 females and 3 males) out of the 18 European wildcats were sampled several times (from two to 6 times). We detected wildcats genetically confirmed as such in elevations up to 2,250 m. In northeastern France, the genetic analyses were in line with the morphological identification: subspecies assignations inferred based on morphological data were confirmed by genetic results. Forty‐two wildcats and 371 domestic cats were thus analyzed thereafter. The material type (blood, hairs, feces), the subspecies, and the geographical area (Pyrenees, northeastern France) were not associated with different proportions of missing data in the consensus genotypes.

### 3.2 Genetic diversity and kinship pattern

We did not detect any significant linkage disequilibrium in any of the populations. The northeastern population of domestic cats showed signs of Hardy–Weinberg disequilibrium for six loci (Fca8, Fca45, Fca96, Fca229, Fca453). The presence of null alleles did not significantly explain these deviations.

Both the subspecies and the geographical area significantly explained the variability observed in our populations (*p* < .001). European wildcats and domestic cats were substantially differentiated with a *F*_ST_ value of 0.17 in the Pyrenees, and 0.15 in northeastern France (see Table 1 for more details on the genetic diversity of the different populations). A significant differentiation between Pyrenean wildcats and northeastern domestic cats (*F*_ST_ = 0.17) on one hand, Pyrenean wildcats and northeastern wildcats (*F*_ST_ = 0.072) on the other hand, was also found, while northeastern domestic cats were, to a lesser extent, differentiated from Pyrenean domestic cats (*F*_ST_ = 0.034). The STRUCTURE analysis confirmed the sharp genetic differentiation between Pyrenean wildcats and northeastern wildcats (Figure S4) with Pyrenean wildcats assigned to one specific cluster whatever the value of *K*. On the contrary, domestic cats from the Pyrenees did not differ from northeastern domestic cats (Figure S4).

**Table 1:** Mean number of alleles (Na), observed heterozygosity (Ho) and expected heterozygosity (He) per locus for each population of wildcats and domestic cats: Pyrenean wildcats (PO wildcats), northeastern wildcats (NE wildcats), Pyrenean domestic cats (PO domestic cats) and northeastern domestic cats (NE domestic cats)

Marker	PO wildcats (*N* = 18)			NE wildcats (*N* = 18)			PO domestic cats (*N* = 18)			NE domestic cats (*N* = 18)		
	Na	Ho	He	Na	Ho	He	Na	Ho	He	Na	Ho	He
Fca8	7	0.78	0.75	7.30	0.76	0.80	6.80	0.85	0.80	8.90	0.76	0.83
Fca45	5	0.67	0.70	6.57	0.78	0.74	7.90	0.71	0.79	9.87	0.78	0.83
Fca58	4	0.33	0.29	5.83	0.59	0.48	6.00	0.90	0.78	6.50	0.59	0.67
Fca96	7	1.00	0.80	8.93	0.39	0.84	5.53	0.45	0.46	6.07	0.39	0.43
Fca124	7	0.88	0.82	7.70	0.76	0.79	6.87	0.85	0.79	7.20	0.76	0.75
Fca126	6	0.67	0.73	6.23	0.49	0.73	9.80	0.77	0.86	5.27	0.49	0.56
Fca577	4	0.64	0.63	5.70	0.70	0.49	6.77	0.69	0.77	7.50	0.70	0.76
Fca668	4	0.82	0.69	6.07	0.83	0.67	5.80	0.71	0.71	9.27	0.83	0.84
Fca675	10	1.00	0.88	10.10	1.00	0.86	10.33	1.00	0.82	9.00	1.00	0.83
Fca26	9	1.00	0.85	12.10	1.00	0.89	10.80	1.00	0.88	13.20	1.00	0.88
Fca069	7	1.00	0.81	9.93	1.00	0.84	9.77	1.00	0.84	11.83	1.00	0.87
Fca075	8	1.00	0.79	10.87	1.00	0.87	10.60	1.00	0.83	10.43	1.00	0.82
Fca105	10	1.00	0.88	11.10	1.00	0.87	8.67	1.00	0.79	10.93	1.00	0.86
Fca149	8	1.00	0.83	8.63	1.00	0.79	8.90	1.00	0.84	12.57	1.00	0.88
Fca201	7	1.00	0.76	9.37	1.00	0.85	10.70	1.00	0.87	9.87	1.00	0.85
Fca220	6	1.00	0.71	9.27	1.00	0.84	9.83	1.00	0.82	10.67	1.00	0.80
Fca229	9	1.00	0.80	11.03	1.00	0.87	7.63	1.00	0.79	9.77	1.00	0.80
Fca293	5	1.00	0.75	7.20	1.00	0.75	8.00	1.00	0.84	10.27	1.00	0.85
Fca310	3	1.00	0.62	4.93	1.00	0.70	7.70	1.00	0.77	8.60	1.00	0.78
Fca441	4	1.00	0.63	5.67	1.00	0.69	8.00	1.00	0.86	8.53	1.00	0.83
Fca453	4	1.00	0.63	6.87	1.00	0.81	7.57	1.00	0.81	8.03	1.00	0.81
Fca678	5	1.00	0.72	4.63	0.95	0.69	7.73	0.95	0.78	6.77	0.95	0.79
Mean	6.32	0.90	0.73	8.00	0.87	0.77	8.26	0.90	0.80	9.14	0.87	0.79
*SD*	2.10	0.18	0.13	2.23	0.19	0.11	1.64	0.15	0.09	2.08	0.19	0.11

Females from the Pyrenean wildcat population showed a *F*_IS_ value close to zero (*F*_IS_ = 0.00028), while males showed a heterozygote excess (*F*_IS_ = −0.09). Wildcat females also presented a higher *F*_IS_ than males in northeastern France but with females being inbred (*F*_IS_ = 0.04) and males at equilibrium (*F*_IS_ = −0.01). On average, the wildcat populations (0.0071 in NE, −0.066 in PO) had lower *F*_IS_ than domestic cat populations (0.062 in NE, 0.026 in PO). In both populations, individuals were poorly related on average (mean = 0.050, *SD* = 0.11 in the Pyrenees; mean = 0.038, *SD* = 0.080 in northeastern France), with *r* ranging from 0 to 0.58 in the Pyrenees and from 0 to 0.52 in northeastern France. In the Pyrenees, in both sexes, related wildcat individuals were not sampled significantly closer together compared to unrelated individuals in the Pyrenees according to the linear mixed model (Figures S5 and S6, *p‐*values > .05). On the contrary, in northeastern France, related females were captured significantly closer together than unrelated females (*F* = 6.88, *df* = 1, *p* = .0095; *r* ^2^ = .033, Figure S5).

### 3.3 Admixture analysis

**Figure 3:**
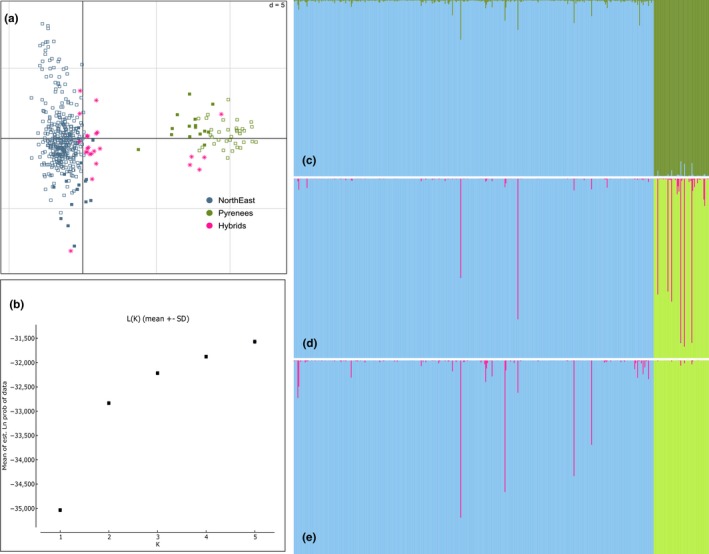
Outputs of the methods used to detect hybrids. (a) Principal component analysis. Pyrenean individuals are represented by empty squares, northeastern individuals by filled squares. The status of individuals has been determined based on the methods used to detect hybrids. Individuals detected as hybrids by at least two methods are represented as such in purple, domestic cats are in blue, European wildcats in green. (b) Likelihood according to the number of clusters considered in STRUCTURE analysis (over all individuals). (c) STRUCTURE output. Domestic cats are represented in blue, and wildcats are in green. (d) NewHybrids output, blue stands for the domestic cluster, green for the wildcat cluster and purple for the hybrid categories (first‐generation backcrosses). F1 and F2 probabilities were very small and are not visible on this illustration. (e) Output from Snapclust. Blue stands for the domestic cluster, green for the wildcat cluster and purple for F1

Both Evanno's method and the PCA showed that our dataset could be partitioned into two clusters: one cluster corresponding to the domestic cats, the other corresponding to the European wildcats (Figure 3).

Only one individual, a domestic cat from NE, was detected as hybrid by all six (“general analysis”—relaxed and conservative, “resamplings”—relaxed and conservative, NewHybrids, Snapclust) approaches (Table 2). Nineteen additional domestic individuals were detected as hybrids by at least one approach. Nine out of these 19 were detected by at least two approaches. No wildcat in NE was detected across all six approaches but six individuals were detected by at least two approaches. Out of these six individuals, only one was a roadkill.

**Table 2:** Probability assignment of all individuals showing signs of hybridization with at least one of the methods

Count	MI	AREA	Sample	NewHybrids						STRUCTURE						Snapclust			Resamplings	
				Catus	Sil	F1	F2	BC_Catus	BC_Sil	Catus	Sil	CI_Catus		CI_Sil		Catus	Sil	F1	%Hybrid_Rel	%Hybid_cons
6	C	NE	B/T	0.45	0.00	0.00	0.01	0.55	0.00	0.78	0.23	0.56	1.00	0.00	0.45	0.19	0.00	0.81	1.00	0.57
4	C	NE	B/T	0.93	0.00	0.00	0.00	0.06	0.00	0.87	0.13	0.59	1.00	0.00	0.41	0.40	0.00	0.60	1.00	0.50
3	C	NE	B/T	0.22	0.00	0.00	0.00	0.78	0.00	0.83	0.17	0.66	0.98	0.02	0.34	0.83	0.00	0.17	1.00	0.43
3	C	NE	B/T	0.95	0.00	0.00	0.00	0.05	0.00	0.88	0.12	0.59	1.00	0.00	0.41	0.32	0.00	0.68	0.83	0.00
2	C	NE	B/T	0.94	0.00	0.00	0.00	0.06	0.00	0.91	0.09	0.66	1.00	0.00	0.34	0.56	0.00	0.44	1.00	0.22
2	C	NE	B/T	0.95	0.00	0.00	0.00	0.05	0.00	0.95	0.05	0.77	1.00	0.00	0.23	1.00	0.00	0.00	0.67	0.00
2	C	NE	B/T	0.98	0.00	0.00	0.00	0.02	0.00	0.95	0.05	0.74	1.00	0.00	0.26	0.89	0.00	0.11	0.67	0.00
2	C	NE	B/T	0.94	0.00	0.00	0.00	0.06	0.00	0.95	0.06	0.70	1.00	0.00	0.30	0.86	0.00	0.14	1.00	0.00
2	C	NE	B/T	0.97	0.00	0.00	0.00	0.03	0.00	0.96	0.04	0.77	1.00	0.00	0.23	0.95	0.00	0.05	1.00	0.00
2	C	NE	B/T	0.99	0.00	0.00	0.00	0.01	0.00	0.97	0.03	0.79	1.00	0.00	0.21	0.81	0.00	0.19	0.71	0.00
1	C	NE	B/T	0.99	0.00	0.00	0.00	0.01	0.00	0.96	0.04	0.77	1.00	0.00	0.23	0.91	0.00	0.09	0.33	0.14
1	C	NE	B/T	0.99	0.00	0.00	0.00	0.01	0.00	0.98	0.02	0.84	1.00	0.00	0.16	1.00	0.00	0.00	0.75	0.00
1	C	NE	B/T	0.99	0.00	0.00	0.00	0.01	0.00	0.97	0.03	0.80	1.00	0.00	0.20	0.97	0.00	0.03	0.00	0.00
1	C	NE	B/T	1.00	0.00	0.00	0.00	0.00	0.00	0.91	0.09	0.72	1.00	0.00	0.29	0.94	0.00	0.06	0.20	0.00
1	C	NE	B/T	1.00	0.00	0.00	0.00	0.00	0.00	0.99	0.01	0.91	1.00	0.00	0.09	0.99	0.00	0.01	0.56	0.00
1	C	NE	B/T	1.00	0.00	0.00	0.00	0.00	0.00	0.99	0.01	0.90	1.00	0.00	0.10	0.99	0.00	0.01	1.00	0.00
1	C	NE	B/T	1.00	0.00	0.00	0.00	0.00	0.00	0.99	0.01	0.96	1.00	0.00	0.04	1.00	0.00	0.00	0.67	0.00
1	C	NE	B/T	1.00	0.00	0.00	0.00	0.00	0.00	0.99	0.01	0.94	1.00	0.00	0.06	1.00	0.00	0.00	0.60	0.00
1	C	NE	B/T	0.99	0.00	0.00	0.00	0.01	0.00	0.96	0.04	0.77	1.00	0.00	0.23	0.92	0.00	0.08	0.33	0.00
1	C	NE	B/T	0.99	0.00	0.00	0.00	0.01	0.00	0.97	0.03	0.81	1.00	0.00	0.19	0.96	0.00	0.04	0.60	0.00
3	S	NE	B/C	0.00	0.08	0.00	0.01	0.00	0.91	0.09	0.92	0.00	0.30	0.70	1.00	0.00	0.97	0.03	0.57	0.00
3	S	NE	B/T	0.00	0.08	0.00	0.00	0.00	0.91	0.08	0.93	0.00	0.28	0.72	1.00	0.00	0.98	0.02	0.50	0.00
2	S	NE	B/T	0.00	0.35	0.00	0.00	0.00	0.64	0.03	0.97	0.00	0.20	0.80	1.00	0.00	1.00	0.00	0.23	0.00
2	S	NE	B/T	0.00	0.06	0.00	0.00	0.00	0.94	0.07	0.93	0.00	0.26	0.74	1.00	0.00	0.99	0.01	0.47	0.00
2	S	NE	B/T	0.00	0.31	0.00	0.00	0.00	0.68	0.03	0.97	0.00	0.20	0.80	1.00	0.00	0.99	0.01	0.17	0.00
1	S	NE	B/T	0.00	0.37	0.00	0.00	0.00	0.63	0.01	0.99	0.00	0.08	0.92	1.00	0.00	1.00	0.00	0.00	0.00
2	C	PO	P	0.98	0.00	0.00	0.00	0.02	0.00	0.97	0.03	0.78	1.00	0.00	0.22	0.90	0.00	0.10	0.53	0.00
1	C	PO	P	0.99	0.00	0.00	0.00	0.01	0.00	0.97	0.03	0.79	1.00	0.00	0.21	0.93	0.00	0.07	0.03	0.00
1	C	PO	P	1.00	0.00	0.00	0.00	0.00	0.00	0.86	0.15	0.68	1.00	0.00	0.32	1.00	0.00	0.00	0.47	0.00

Note

The first column indicates the number of approaches that detect the individual as hybrid. The next three columns indicate the species to which individuals were assigned based on their morphology (Morphological identification, MI, C for Catus and S for Silvestris), the geographical area where the individual was sampled (NE: northeastern, PO: Pyrénées Orientales) and the type of sample (B/T for live‐trapped, B/C for roadkills, P for hairs). Probabilities of assignment to the domestic cluster (Fsc), the European wildcat cluster (Fss), F1 hybrids (F1), first‐generation backcrosses (BC_Fsc for F1 × Fsc, BC_Fss for F1 × Fss) computed by NewHybrids, Structure and Snapclust are given in the subsequent columns. The columns CI_Fsc and CI_Fss falling under STRUCTURE correspond to the 95% credibility intervals associated with each mean probability. The %hybrid column indicates how often the individual has been detected as hybrid with the resampling procedure (relaxed and conservative approach)

In the PO, no wildcat presented signs of hybridization across methods. In the domestic cat population, three individuals showed signs of hybridization. These signs were detected by a maximum of two methods, neither NewHybrids, Snapclust or the conservative approaches detected these individuals as hybrids.

Consistently with these results, the confidence interval obtained with the resampling procedure systematically included zero for the PO wildcat population, whatever the approach (CI_95_: 0–0 with the conservative approach; CI_95_: 0–8.61 with the relaxed approach). Two individuals were detected in a minority of the resamplings as hybrids, one of them appeared close to the domestic cluster in the PCA (Figure 3). These individuals were assigned to the wildcat cluster with *q‐*values superior than 0.99 in the general analysis. The confidence interval obtained for the PO domestic population also included zero but only with the conservative approach (CI_95_: 0–1.31). With the relaxed approach, the mean rate of hybridization in the PO domestic population reached 22.56 (CI_95_: 9.52–38.10). In northeastern France, the average rate of hybridization varied between 1.17 (CI_95_: 0–6.37) with the conservative approach and 13.73 (CI_95_: 5.71–22.21) with the relaxed approach in the domestic population. The NE wildcat rate of hybridization varied between 1.19 with the conservative approach (CI_95_: 0–3.04) and 9.52 with the relaxed approach (CI_95_: 4.76–20.6) on average.

The ESS values of the BayesAss analyses were systematically higher than 200. We did not detect any significant gene flow from domestic cats to wildcats (*m* = 0.0148–CI_95_: 0–0.042) as well as from wildcats to domestic cats (*m* = 0.0160–CI_95_: 0–0.046) in the Pyrenean population. Similar results were found in northeastern France: no flow from domestic cats to wildcats (*m* = 0.0009–CI_95_: 0–0.0021) or from European wildcats to domestic cats (*m* = 0.0076–CI_95_: 0–0.017).

## 4 DISCUSSION

Our study confirmed the presence of wildcats in the French Pyrenees within a large area of the nature reserves of Nohèdes and Jujols up to 2,430 m. Moreover, our study has provided the first genetic characterization of a local population of the French Pyrenean wildcats, despite their presence being acknowledged since 1993 (Say et al., 2012).

### 4.1 Low genetic diversity pattern in Pyrenean wildcats

The Pyrenean wildcats showed values of genetic diversity lower than other wildcat or domestic cat populations in France and in Europe (6.32–4.84 when considering alleles with frequencies over 5%—alleles per locus on average in the Pyrenean wildcat population while between 3 and 11.8 can be found in the literature with rare populations below 6; Germain et al., 2008, 2009; O'Brien et al., 2009; Oliveira et al., 2007; Say et al., 2012). This low genetic diversity may suggest that wildcats in the Pyrenees are isolated from other wildcat populations or have gone through some bottleneck in the past. This hypothesis would be consistent with the distribution gap of the wildcat population suggested by previous authors (O'Brien et al., 2008; Say et al., 2012) in France as well as with the substantial differentiation observed between Pyrenean and northeastern wildcats in our study (*F*_ST_ = 0.072). In addition, the mountainous environment may reduce connectivity and associated gene flow, which would result in low genetic diversity in the PO wildcat population. Additional studies aiming at describing individual movements (either by radio tracking or more extensive noninvasive sampling) should be considered in the future to assess resulting genetic flow. Finally, French Pyrenean wildcats may be at the colonization front of a wider European wildcat population. First, PO and NE populations may correspond to two extremities of a continuum. Their divergence would result from isolation‐by‐distance (IBD, Wright, 1943); such a cline having been described in the northeastern wildcat population (Say et al., 2012; Würstlin, Segelbacher, Streif, & Kohnen, 2016). Studies on the distribution of the European wildcat in France do not support the existence of such continuum so far (Say et al., 2012). However, the presence of wildcats may have been missed because, for example, of different sampling effort or to local species density. Alternatively, the PO wildcat population may originate from the Iberian population, which is genetically differentiated from the German population (to which NE is connected, Mattucci et al., 2016) to an extent that is similar to what we observed between PO and NE (0.10 in Mattucci et al., 2016 between Fs4 and Fs5 wildcat population vs. 0.072 in this study). The PO population would thus belong to a different biogeographical unit than the NE population. This would suppose that wildcats can cross the Pyrenees, which is consistent with the maximal elevations (2,430 m) at which we collected feces, and the identification several times through camera traps of wildcats at high altitude in winter within harsh conditions (snow).

### 4.2 Hybridization patterns

Both in the Pyrenees and in northeastern France, no hybrid presented *q*‐values consistent with F1 (always above 0.75). In both geographical areas, hybrids were detected in larger proportions within domestic cats' populations than within wildcats (in northeastern France; we found no hybrids in the PO wildcats). In northeastern France, this pattern is consistent with the sex‐biased spatial organization of the wildcat population, where males are at the periphery of the forest and females at the core, which was proposed to promote hybridization between wildcat males and domestic cat females (Beugin et al., 2016). Such sex‐biased hybridization would result in a higher number of hybrids in domestic populations, both because female domestic cats are generally philopatric or do not disperse far (Liberg, 1980) and territoriality and aggressive interactions within wildcats (Piechocki, 1990) may lead to domestic cats being expelled from wildcat habitat. Despite it has been often reported a nonsignificant difference in body weight between domestic and wildcats (Beugin et al., 2016), domestic male cats might not be able to gain and maintain territories in a wild environment where wildcats are present as competitors (Sunquist & Sunquist, 2002). This is probably due to the fact that domestication has influenced some characteristics of the domestic cat (Cameron‐Beaumont, Lowe, & Bradshaw, 2002; Driscoll, Macdonald, & O'Brien, 2009; Mattucci et al., 2019; Wilkins, Wrangham, & Fitch, 2014), first of all the dependence on food distributed by humans, that has made it less competitive than the ancestor and other wild related subspecies. A low abundance of rodents and/or competition with other carnivore species (Gil‐Sànchez et al., 2015) such as the red fox (*Vulpes vulpes*)—of which populations are increasing in several European countries (Chautan, Pontier, & Artois, 2000; Goszczyński, Misiorowska, & Juszko, 2008)—may make domestic cats even more heavily dependent on food distributed by humans.

In the Pyrenees, we did not detect a similar sex‐biased spatial organization in the wildcat population. However, the opportunistic collection of feces may not provide enough information compared to trapping, to assess the spatial organization pattern of this wildcat population. More systematic noninvasive sampling should be conducted in the future to better characterize the home ranges of wildcats according to their sex in the Pyrenees.

As expected, hybridization seemed more frequent in northeastern France than in the Pyrenees, which may directly result from the large and continuous forest in the Pyrenees, reducing the contact between wild and domestic cats. In northeastern France, the average rate of hybridization (0%–9.52%) observed in wildcats was, in accordance with Beugin et al. (2016), lower than rates previously reported by studies in the same area (25% on average, Germain et al., 2008, 2009; O'Brien et al., 2009; Say et al., 2012). Such variation between studies may be partly due to inadequate choice of markers and/or sampling design as discussed in Steyer, Tiesmeyer, Muños‐Fuentes, and Nowak (2018). The absence of significant gene flow between the two subspecies using BayesAss is consistent with the results obtained with the conservative method, which all led to low rates of hybridization (below 2% on average all populations confounded). The actual rate of hybridization may thus be better captured by the conservative approach. Nevertheless, it remains useful to work both with the relaxed and conservative approach, especially when working with microsatellite markers, which are less efficient than SNP markers to detect hybrids (Steyer et al., 2018).

In the Pyrenees, the absence of clear hybridization in the wildcat population may reflect difficulties for hybrids to survive in the wildcat habitat. Mountainous habitats are indeed expected to be rougher than plains and may prevent hybrids' survival. More generally, the low rates of hybridization found in this study suggest the existence of behavioral or/and ecological barriers both in the Pyrenees and in northeastern France preventing the reproduction of hybrids with parental individuals. Hartmann, Steyer, Kraus, Segelbacher, and Nowak (2013) showed that gene flows in wildcats in Germany are disrupted by rivers or highways. Small roads and small streams being present in our areas of study, further work will be necessary to precise their role in preventing gene flows among wildcat populations and between subspecies, and to assess whether other types of barriers exist and to what extent they are similar in both areas.

However, it is important to be cautious with this comparison as the sampling method used for the two sites were different (trapped and some road‐killed individuals were used for the NE population whereas opportunistically found feces were used for the PO population). Nevertheless, the bias should not be too high, as the capture of individuals and fecal collect were realized in assumed wildcat habitats (forest region) for both sites. Furthermore, contrary to Germain et al. (2008, 2009), who found that hybrids tend to live in intermediary environments, for the NE population, wildcats that presented signs of hybridization were almost found within the trapped individuals.

### 4.3 Variability in the dispersal pattern of wildcats

Interestingly, males and females in close proximity in the Pyrenees are not kin related suggesting that both males and females disperse in this continuous forest landscape, that is, related females do not tend to remain in the same area contrary to the wildcat population of northeastern France. The dispersal pattern may directly reflect the level of food resource availability. In fragmented environments as observed in northeastern France, with forest alternating with field crops, large areas rich in resources are available for wildcats (Lozano, Virgós, Malo, Huertas, & Casanovas, 2003; Silva, Kilshaw, Johnson, MacDonald, & Rosalino, 2013). Food distribution has indeed been suggested to be the major determinant of species spatial distribution in carnivores (Macdonald, 1983). The importance of resource distribution on the spacing pattern of wildcat females has already been proposed (Sarmento, Cruz, Eira, & Fonseca, 2009; Stahl, Artois, & Aubert, 1988) and was supported by the study in northeastern France (Beugin et al., 2016). Thus, although the European wildcat is acknowledged to live solitarily (Biró, Szemethy, & Heltai, 2004; Corbett, 1979), its dispersal pattern may show more variability than has been described up to now.

### 4.4 Perspectives

Our results have added novel information to the European wildcat population structure in France. They provided further information about the relationship between environmental conditions and hybridization risks in French wildcat populations. Further genetic analyses—combining microsatellite and SNP markers, which are more powerful in detecting patterns and histories of admixtures (Mattucci et al., 2019; Nussberger, Greminger, Grossen, Keller, & Wandeler, 2013), as well as mitochondrial and Y chromosome markers for evaluating possible asymmetry in hybridization (hybridization between female wildcats and male domestic cats or vice versa; Hertwig et al., 2009; Rhymer & Simberloff, 1996)—should lead to a deeper insight into the hybridization and long‐term patterns of French wildcat populations.

Further investigation should focus on the spatial distribution of French wildcats—in particular we need to confirm whether the French Pyrenean population is isolated from the main area of distribution of wildcats in France but connected to the Spanish Pyrenean wildcat population. Depending upon the answer, the usefulness of wildlife corridors to enhance connectivity between the different wildcat populations should be addressed to ensure the long‐term viability of the French Pyrenean wildcat population.

Finally, conservation strategies of wildcats should take into account local habitat features such as the existence of a fragmented or continuous forest environment and the presence of agricultural fields (Jerosch, Kramer‐Schadt, Götz, & Roth, 2018). A better knowledge of how different landscape features impact behavioral interactions (e.g., assortative mating) between wildcats, domestic cats and their hybrids is required to better understand variations in hybridization rates and their consequences for European wildcats' conservation.

## CONFLICT OF INTEREST

None declared.

## AUTHORS CONTRIBUTION

DP conceptualized, designed the study, and acquired funding. GL, OS, M‐PB, and DP performed samplings on the field. GQ carried out the genotyping of microsatellite markers. M‐PB performed the formal analysis of the data. M‐PB, EN, and DP wrote the manuscript. All authors read and annotated the final version of the manuscript.

## Data Availability

All genotypes and locations will be available upon acceptance of the manuscript on DRYAD: URL: https://datadryad.org/stash/share/RQtTtqj8vwTw23ohXj1_DJbo9QqMfnQcEle67pZ6toE; https://doi.org/10.5061/dryad.xksn02vbh.
