# Supplementary material for: Hybridization between Felis silvestris silvestris and Felis silvestris catus in two contrasted environments in France
Source: Ecol Evol. 2019 Dec 2;10(1):263–76. doi: 10.1002/ece3.5892 (PMC6972816; doi:10.1002/ece3.5892)

**Hybridization between *Felis silvestris silvestris* and *Felis silvestris catus* in two contrasted environments in France**

BEUGIN Marie-Pauline^1,2^, SALVADOR Olivier^3^, LEBLANC Guillaume, QUENEY Guillaume^2^, NATOLI Eugenia^4^ and PONTIER Dominique^1^

SUPPLEMENTARY MATERIAL

Table S1:

Genotyping protocol and primers for the 31 microsatellite markers. The columns NE and PO precise whether the marker was genotyped and used to analyze the Northeastern population and the Pyrenean population respectively.

| Marker | Primers | Dye | Annealing | Multiplex | NE | PO |
| --- | --- | --- | --- | --- | --- | --- |
| ZFXY | F: AAGTTTACACAACCACCTGG  R: CACAGAATTTACACTTGTGCA | NED | 51°C | 2 & 3 | Yes | Yes |
| F37 | F: CGCCTTTCTCACATTACCAT  R: CACTGACAGATCTGATCCTG | PET | 55°C | 2 | No | Yes |
| Fca8 | F: ACTGTAAATTTCTGAGCTGGCC  R: TGACAGACTGTTCTGGGTATGG | 6FAM | 56°C | 1 | No | Yes |
| Fca023 | F: CAGTTCCTTTTTCTCAAGATTGC  R: GCAACTCTTAATCAAGATTCCATT | 6FAM | 52°C | 2 | No | Yes |
| Fca024 | F: CCCAGCTTTGTCTCTTACTGTG  R:  CATCCTCCCCTAATGCCC | PET | 56°C | 1 | No | Yes |
| Fca031 | F: GCCAGGGACCTTTAGTTAGATT  R: GCCCTTGGAACTATTAAAACCA | 6FAM | 55°C | 2 | No | Yes |
| Fca43 | F: GAGCCACCCTAGCACATATACC  R: AGACGGGATTGCATGAAAAG | NED | 53°C | 1 | No | Yes |
| Fca45 | F: TGAAGAAAAGAATCAGGCTGTG  R: GTATGAGCATCTCTGTGTTCGTG | VIC | 54°C | 1 | Yes | Yes |
| Fca58 | F: CATCCCTGACTAGCCTGAGC  R: GTGAAGAAAGCTGGTGTGCA | 6FAM | 57°C | 1 | Yes | Yes |

| Marker | Primers | Dye | Annealing | Multiplex | NE | PO |
| --- | --- | --- | --- | --- | --- | --- |
| Fca77 | F: GGCACCTATAACTACCAGTGTGA  R: ATCTCTGGGGAAATAAATTTTGG | VIC | 51°C | 2 | No | Yes |
| Fca078 | F: TGAACTGAAGTCAGATGCTTAACC  R: CGGAATCAGCTATTTTTACGG | 6FAM | 52°C | 1 | No | Yes |
| Fca085 | F: CTGTACATTTCTCTTCCCATTGC  R: CCCCTACTGGGTGCACTG | NED | 55°C | 2 | No | Yes |
| Fca96 | F: CACGCCAAACTCTATGCTGA  R: CAATGTGCCGTCCAAGAAC | VIC | 55°C | 1 | Yes | Yes |
| Fca124 | F: CCATTCCCTCCCTGTCTGTA  R: GCCTCAAGCCTCATTGCTAC | VIC | 57°C | 1 | Yes | Yes |
| Fca126 | F: GCCCCTGATACCCTGAATG  R: CTATCCTTGCTGGCTGAAGG | PET | 56°C | 2 | Yes | Yes |
| Fca547 | F: TGGTCATACAGGTGACAAAACA  R: CTGACAGTATGGAGCCTGCA | VIC | 55°C | 2 | No | Yes |
| Fca577 | F: AAGCAGCATGTATTCACACA  R: GTGTCCCAGAGAGTTATCAGGG | NED | 54°C | 1 | Yes | Yes |
| Fca668 | F: AATGGCTGAGACATCAAGTGG  R: AGAGCAGCCCAAAAAAGTCA | NED | 56°C | 1 | Yes | Yes |
| Fca675 | F: CTATTGCTTCCAGCCAGTCC  R: GCCATGGACAAGAGACTGGT | 6FAM | 57°C | 2 | Yes | Yes |
| Marker | Primers | Dye | Annealing | Multiplex | NE | PO |
| Fca26 | F: GGAGCCCTTAGAGTCATGCA  R: TGTACACGCACCAAAAACAA | PET | 54°C | 1 & 3 | Yes | Yes |
| Fca069 | F: AATCACTCATGCACGAATGC  R: AATTTAACGTTAGGCTTTTTGCC | 6FAM | 52°C | 3 | Yes | Yes |
| Fca075 | F: ATGCTAATCAGTGGCATTTGG  R: GAACAAAAATTCCAGACGTGC | NED | 53°C | 3 | Yes | Yes |
| Fca105 | F: TTGACCCTCATACCTTCTTTGG  R: TGGGAGAATAAATTTGCAAAGC | 6FAM | 52°C | 3 | Yes | Yes |
| Fca149 | F: CCTATCAAAGTTCTCACCAAATCA  R: GTCTCACCATGTGTGGGATG | PET | 53°C | 3 | Yes | Yes |
| Fca201 | F: TCTGCAGGACCAGTCAGATG  R: AGCATACACAAATTGATGCTGG | 6FAM | 53°C | 3 | Yes | Yes |
| Fca220 | F: CGATGGAAATTGTATCCATGG  R: GAATGAAGGCAGTCACAAACTG | NED | 53°C | 3 | Yes | Yes |
| Fca229 | F: CAAACTGACAAGCTTAGAGGGC  R: GCCAATGAATGCTGCCTAAC | VIC | 57°C | 3 | Yes | Yes |
| Fca293 | F: GATGGCCCAAAAGCACAC  R: CCCACATCTTGTCAACAACG | NED | 55°C | 3 | Yes | Yes |
| Fca310 | F: TTAATTGTATCCCAAGTGGTCA  R: TAATGCTGCAATGTAGGGCA | VIC | 52°C | 3 | Yes | Yes |
| Marker | Primers | Dye | Annealing | Multiplex | NE | PO |
| Fca441 | F: ATCGGTAGGTAGGTAGATATAG  R: GCTTGCTTCAAAATTTTCAC | PET | 49°C | 3 | Yes | Yes |
| Fca453 | F: AATTCTGAGAACAAGCTGAGGG  R: ATCCTCTATGGCAGGACTTTG | VIC | 56°C | 3 | Yes | Yes |
| Fca678 | F: TCCCTCAGCAATCTCCAGAA  R: GAGGGAGCTAGCTGAAATTGTT | 6FAM | 57°C | 3 | Yes | Yes |

DNA extraction was performed using a purification column kit (Nucleospin 96 Tissue kit, Macherey-Nagel) following the manufacturer protocol. PCR reactions were performed step-by-step following a unidirectional workflow starting in a clear room with positive air pressure where sensitive reagents, enzymes and primers, were prepared. DNA and reagents were then assembled in a pre-PCR room. PCR amplifications were made in 96-well microplates in a post-PCR area with negative air pressure. The PCR reaction occurred in a final volume of 10µl that contained 5µl of Mastermix Taq polymerase (Type-it, QIAGEN), 1.35µl of primer pairs at a final concentration between 0.08 and 0.6µM, and 30ng of DNA. Each pair of primers was coupled with a fluorescent dye. The reaction started with a denaturation step at 95°C for five minutes. This step was followed by thirty PCR cycles (denaturation step = 95°C, 30s; annealing step = 55.9°C, 90s; elongation step = 72°C, 30s) and a final elongation step at 60°C during 30 minutes. PCR products were resolved on a capillary sequencer ABI PRISM 3130 XL (Applied Biosystem) under denaturing conditions (formamide) and an internal size marker in one migration for each multiplex. All these steps were performed using filtered tips. The electrophoregrams were analyzed using GENEMAPPER 4.1 (Applied Biosystem/Life Technologies) twice independently. Ambiguous loci were classed as missing data.

**Figure S1:**

Difference between the conservative and the relaxed approach for the detection of hybrids. Black spots represent individual probabilities of assignment obtained with STRUCTURE and vertical bars represent the 90% credibility interval. We can see that more individuals are detected as hybrids with the relaxed method. The overall approach to detect hybrids is represented at the bottom of the figure.


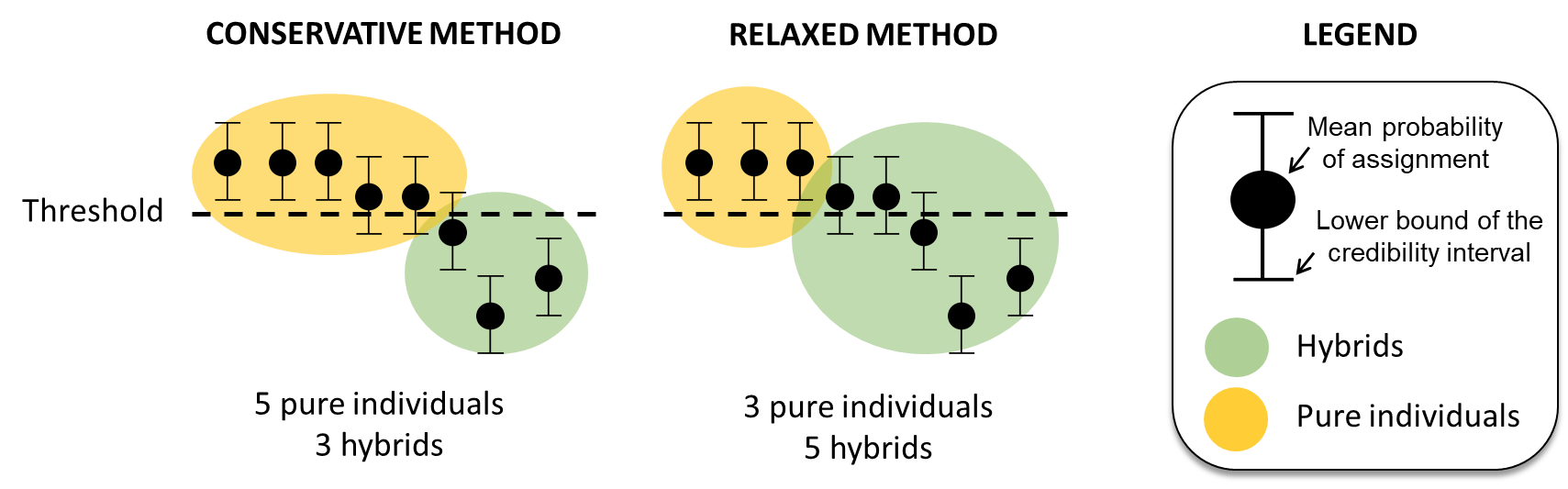


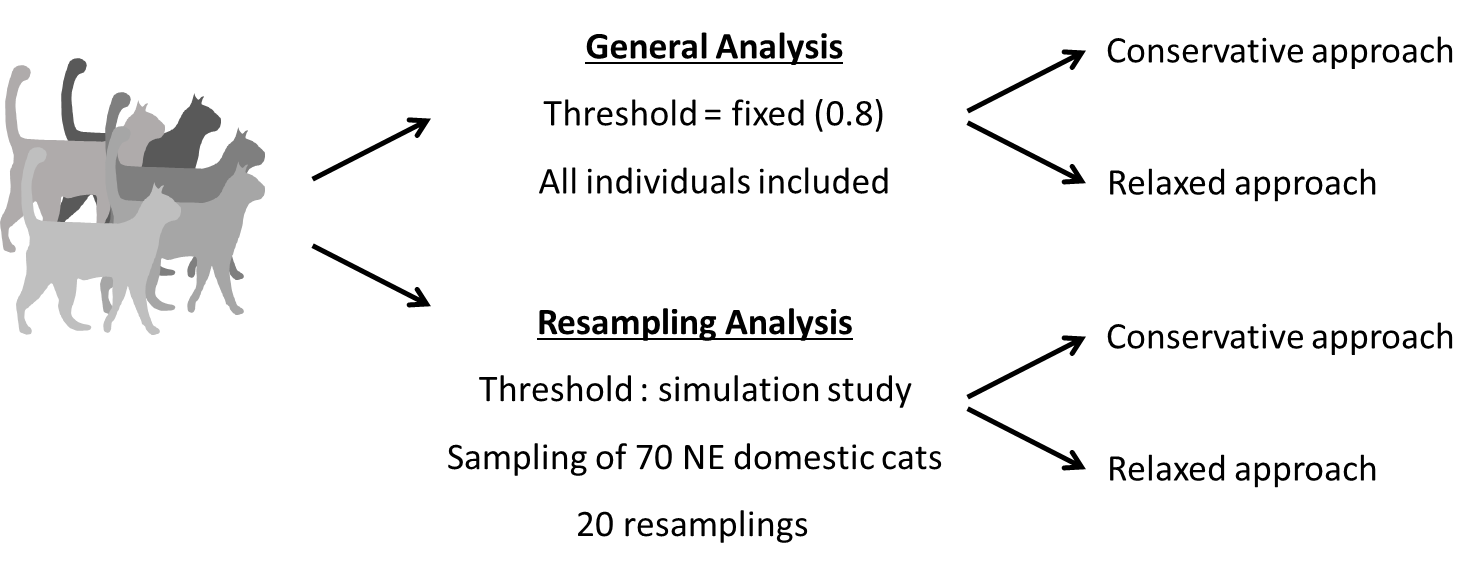


Figure S2:

Distribution of the quality indexes of the samples included in the analyses.

**
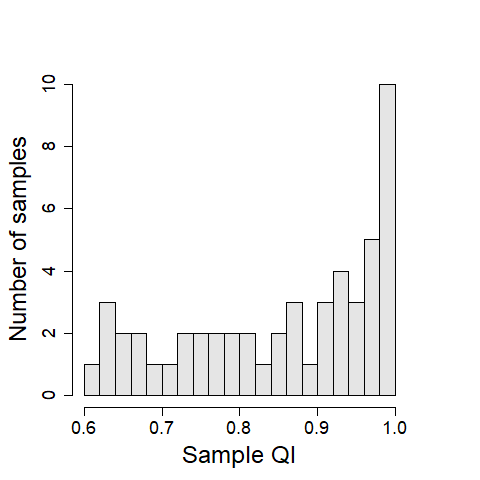
**

Figure S3:

STRUCTURE assignment probabilities to 2 to 5 clusters *K* in wildcats (on the left), domestic cats (on the right), based on 22 microsatellite markers. On each plot, the vertical black line represents the limit between samples originating Northeastern France (NE) and Pyrenees (PO). Each cluster is represented by a different color.


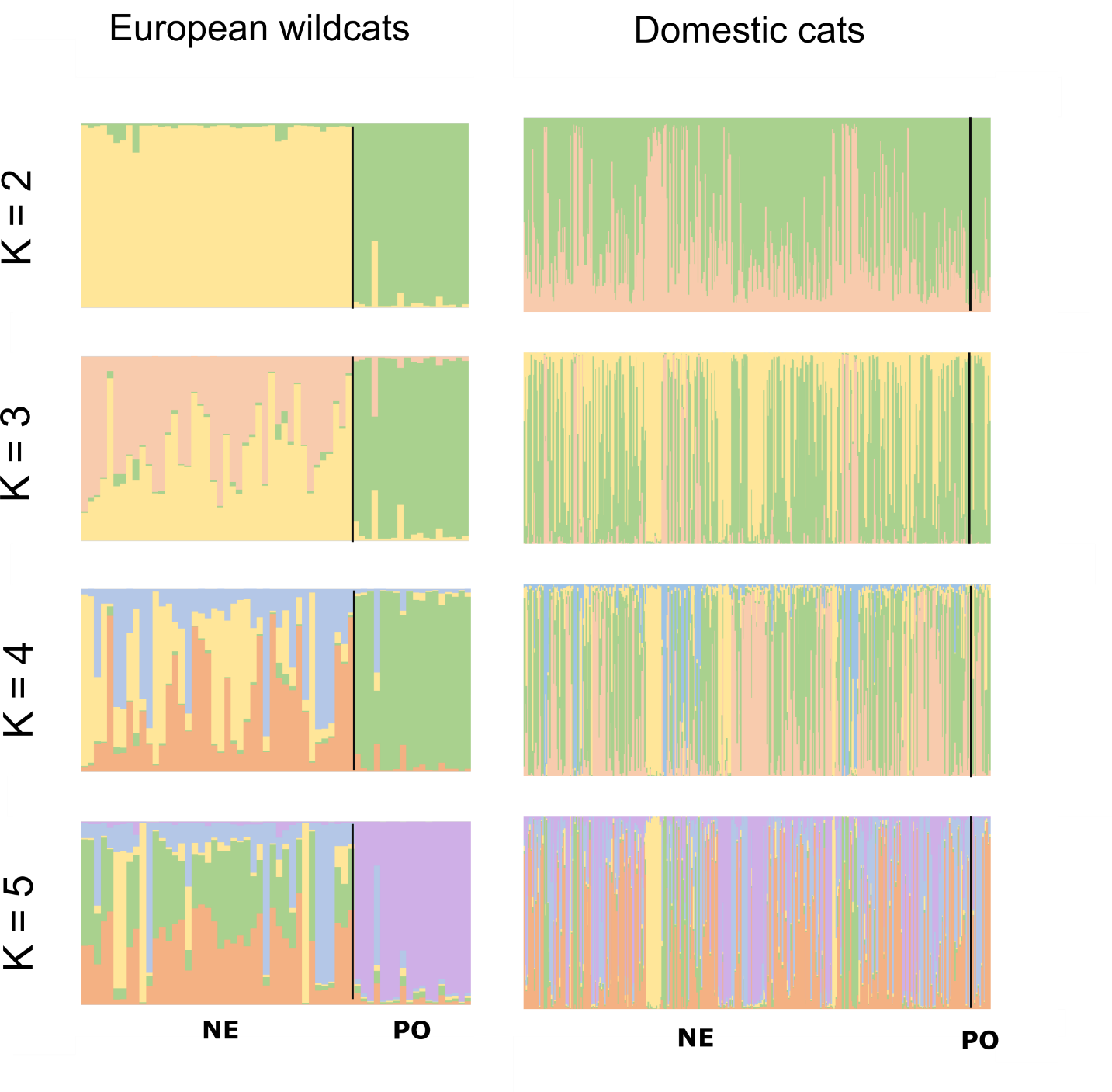


Figure S4:

Locations of the samples according to the sex of individuals in Pyrenees. Females are indicated in orange and males in blue.The grey areas correspond to the zones covered by the nature reserves of Jujols and Nohèdes.

**
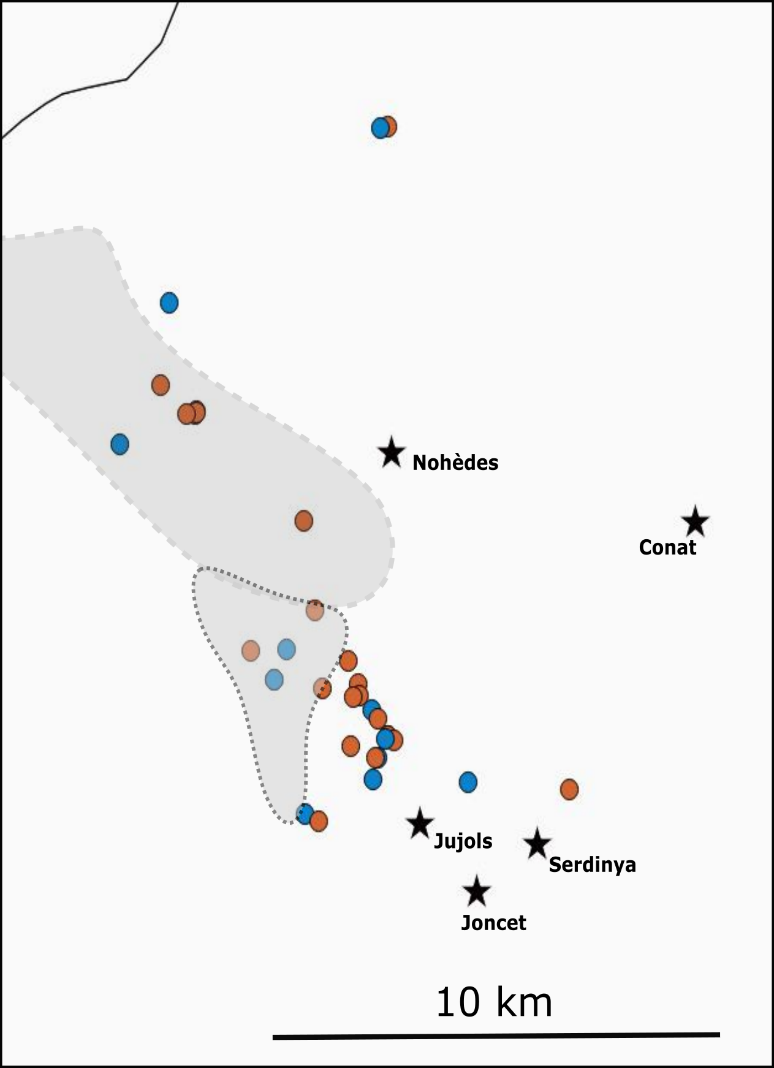
**

Figure S5:

Linear regression and 95% confidence interval of the pairwise distance between trapping location /fresh feces samples according to the coefficient of relationship. A. in Northeastern France and B. in the Pyrenees.


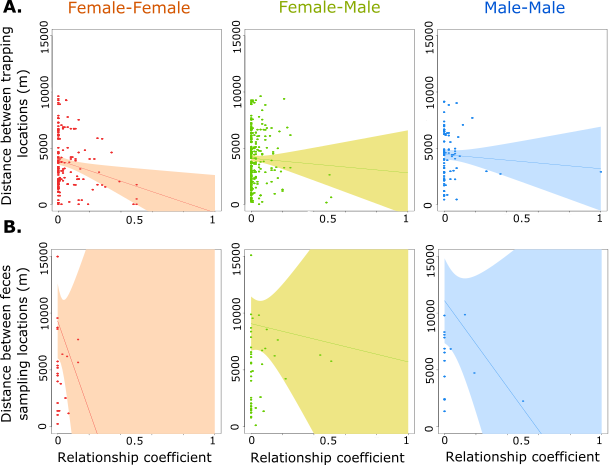

Supplement: Supplementary file 1 [file ECE3-10-263-s001.docx]
